# Loss of IL-10 Promotes Differentiation of Microglia to a M1 Phenotype

**DOI:** 10.3389/fncel.2019.00430

**Published:** 2019-10-09

**Authors:** Björn Laffer, Dirk Bauer, Susanne Wasmuth, Martin Busch, Tida Viola Jalilvand, Solon Thanos, Gerd Meyer zu Hörste, Karin Loser, Thomas Langmann, Arnd Heiligenhaus, Maren Kasper

**Affiliations:** ^1^Department of Ophthalmology and Ophtha-Lab at St. Franziskus-Hospital, Münster, Germany; ^2^Department of Ophthalmology, University of Duisburg-Essen, Essen, Germany; ^3^Department of Experimental Ophthalmology, Westphalian Wilhelms University of Münster, Münster, Germany; ^4^Department of Neurology with Institute of Translational Neurology, University Hospital Münster, Münster, Germany; ^5^Department of Dermatology – Experimental Dermatology and Immunobiology of the Skin, University of Münster, Münster, Germany; ^6^Laboratory for Experimental Immunology of the Eye, Department of Ophthalmology, University of Cologne, Faculty of Medicine and University Hospital Cologne, Cologne, Germany; ^7^University of Duisburg-Essen, Essen, Germany

**Keywords:** cytokines, phenotype, interleukin-10, microglia, M1/2 polarization, lipopolysaccharide

## Abstract

Microglia represent the primary resident immune cells of the central nervous system (CNS) and modulate local immune responses. Depending on their physiological functions, microglia can be classified into pro- (M1) and anti-inflammatory (M2) phenotype. Interleukin (IL)-10 is an important modulator of neuronal homeostasis, with anti-inflammatory and neuroprotective functions, and can be released by microglia. Here, we investigated how IL-10 deficiency affected the M1/2 polarization of primary microglia upon lipopolysaccharide (LPS) stimulation *in vitro*. Microglia phenotypes were analyzed via flow cytometry. Cytokine and chemokine secretion were examined by ELISA and bead-based multiplex LEGENDplex^TM^. Our results showed that genetic depletion of IL-10 led to elevated M1 like phenotype (CD86+ CD206−) under pro-inflammatory conditions associated with increased frequency of IL-6+, TNF-α+ cells and enhanced release of several pro-inflammatory chemokines. Absence of IL-10 led to an attenuated M2 like phenotype (CD86− CD206+) and a reduced secretion of TGF-β1 upon LPS stimulation. In conclusion, IL-10 deficiency may promote the polarization of microglia into M1-prone phenotype under pro-inflammatory conditions.

## Introduction

Microglia are yolk sac-derived myeloid lineage cells (Kierdorf et al., 2013) that are commonly defined as innate immune cells in the CNS. Under homeostatic conditions, predominantly ramified and resting microglia screen their microenvironment to detect injury or infection and act to surveil the CNS by removing cell debris and contributing to neuronal plasticity (Davalos et al., 2005; Nimmerjahn et al., 2005).

Under inflammatory conditions, microglia are the main local producers of cytokines in neuronal tissues (Hanisch, 2002). This surveillant/non-polarized phenotype is also known as M0, which describes non-activated microglia (Ohsawa and Kohsaka, 2011). They assume an amoeboid shape, proliferate, and migrate to the site of damage (Kreutzberg, 1996; Davalos et al., 2005). Upon disturbance of tissue homeostasis or injury, microglia became activated. Owing to their capability to switch between pro- (M1) and anti-inflammatory/neuroprotective (M2) phenotypes, they can modulate local immune responses depending on the environment, similar to the macrophage M1/2 classification (Martinez et al., 2009; Fernandes et al., 2014). Microglial activation has been characterized by a number of phenotypes classically described for macrophages (Gordon, 2003).

The M1 phenotype (classical, activated microglia) can be induced by LPS or IFN-γ (Hanisch, 2013; Branchi et al., 2014; Orihuela et al., 2016), which is associated with increased production of proinflammatory cytokines/chemokines such as TNF-α, IL-1β, and CC chemokine ligand CCL2, as well as matrix metalloproteinases (MMPs) and reactive oxygen/nitrogen species (ROS/RNS), among others (Durafourt et al., 2012). Moreover, this neurotoxic phenotype also upregulates high levels of MHC class-II and costimulatory proteins such as CD86 and CD40 (Kalkman and Feuerbach, 2016). The M2 phenotype (alternative, activated microglia) is related to resolving damage (Chhor et al., 2013) and can mainly be induced by IL-4 or IL-13 (Martinez et al., 2008). M2 microglia may include several subtypes, such as M2a (wound-healing and anti-inflammatory, expressing CD206, Fizz-1, Arg1, Ym1), M2b (inflammation modulatory, expressing IL-10, COX2), and M2c (immunosuppressive, expressing CD163) (Franco and Fernandez-Suarez, 2015; Du et al., 2017). There is increasing evidence that microglia do not show a constant differentiation to an M1 or M2 phenotype, but rather to several phenotypes with high plasticity. Kumar et al. described a mixed transitional phenotype called Mtran, coexpressing M1 markers (iNOS and IL-12) and M2 markers (TGF-β and Arg1) (Kumar et al., 2016).

Interleukin-10 is a potent anti-inflammatory cytokine that is produced by both human and murine microglia when these cells are stimulated by LPS and/or IFN-γ (Mizuno et al., 1994; Williams et al., 1996; Lee et al., 2002; Seo et al., 2004). IL-10 is an important modulator of glial activation, maintaining the balance between pro- and anti-inflammatory cytokine levels in the CNS. IL-10 is also an important mediator of the crosstalk between microglia, astrocytes, and neurons in the CNS and may be involved in anti-inflammatory (Cianciulli et al., 2015) and neuroprotective effects (Lobo-Silva et al., 2016). Furthermore, it promotes cell survival by expressing anti-apoptotic factors Bcl-2 and Bcl-xl and attenuating caspase 3 activity (Moore et al., 2001).

Administration of IL-10 protected astrocytes from excessive inflammation by inhibiting the production of pro-inflammatory cytokines (Balasingam and Yong, 1996; Ledeboer et al., 2002) and potentiating the production of TGF-β by astrocytes (Norden et al., 2014). Furthermore, IL-10 suppressed the production of pro-inflammatory mediators such as IL-1β, TNF-α, and iNOS and reduced MHC class-II expression *in vitro* (Aloisi et al., 1999; Ledeboer et al., 2000).

Murine and rat models of EAE are well-established models resembling the pathology of MS in humans. There is evidence from these rodent studies that IL-10 plays an important role in onset, severity, and progression of neuronal inflammatory diseases as shown for EAE. IL-10 KO mice developed a stronger pro-inflammatory T cell-mediated immune response with more severe EAE and accelerated disease progression compared to WT mice (Samoilova et al., 1998; Anderson et al., 2004). Moreover, human IL-10 (hIL-10) transgenic mice overexpressing IL-10 were highly resistant to EAE (Cua et al., 1999). This effect was mediated by the suppression of Th1 cells and abolished after systemic administration of anti-IL-10 antibody, showing that resistance to disease development was IL-10-dependent (Bettelli et al., 1998). IL-10-mediated suppression of EAE was further shown by administering low IL-10 concentrations via the nasal route, which are associated with decreased microglial activation, T-cell proliferation, and IFN-γ secretion (Xiao et al., 1998).

These previous studies demonstrate that treatment with IL-10 plays an important role in modulating inflammatory processes in CNS diseases. Here, we studied the role of IL-10 on the M1/2 phenotype of microglia by isolating brain-derived microglia from WT and IL-10 KO mice and analyzing their cytokine/chemokine response to pro-inflammatory culture conditions.

## Materials and Methods

### Mice

C57BL/6J WT (Charles River Laboratories, Wilmington, DE, United States) and C57BL/6J IL-10 knock-out (IL-10 KO) mice (B6.129P2-Il10tm1Cgn/J; Jackson Laboratories, Bar Harbor, ME, United States) (Kuhn et al., 1993) were housed in standard animal rooms under a 12-h light/dark cycle with food and water provided *ad libitum*. Mice were bred with heterozygote mice and selected for the experiments according to genetic homogeneity.

### Isolation of Primary Microglia

Primary brain derived microglia from WT and IL-10 KO mice (2–14 days postnatally, female and male) were isolated via magnetic cell sorting. To perform one experiment 5 mice pubs were used per genotype. In brief, postnatal mice were decapitated, brains were isolated and transferred into ice-cold PBS containing 1% BSA (Carl Roth, Karlsruhe Germany). Brains were minced and enzymatically digested by using Neural Tissue Dissociation Kit – Postnatal Neurons (Miltenyi Biotec, Bergisch Gladbach, Germany). After removing myelin using Myelin Removal Beads II (Miltenyi Biotec), CD11b-positive cells were positively selected by magnetic separation using CD11b (Microglia) MicroBeads, human and mouse (Miltenyi Biotec), according to the manufacturer’s instructions. In total 19 experiments were performed. An average yield of 1.5 ± 0.15 × 10^6^ CD11b+ cells/mouse brain from WT and 1.6 ± 0.17 × 10^6^ CD11b+ cells/mouse brain from IL-10 KO mice were isolated.

### Cell Culture

Primary CD11b-positive brain cells were cultured in 6-well plates (4 × 10^5^/well) (TPP, Trasadingen, Switzerland) or on 8-well chamber slides (3 × 10^4^/well) (Merck Millipore, Darmstadt, Germany). Cells were cultivated in DMEM/F12 medium (Biochrom, Berlin, Germany) supplemented with 10% fetal calf serum (FCS; Biochrom), 1% penicillin/streptomycin (PAA Laboratories, Pasching, Austria), and 1% amphotericin B (VWR International, Darmstadt, Germany), and based on other studies (Devarajan et al., 2014; Scheiblich et al., 2017; Voet et al., 2018) 10% L929 cell (murine fibroblast line, ATCC^®^ CCL-1TM) conditioned medium as a source of growth factors containing 100 pg/ml M-CSF. The M-CSF concentration in L929 supernatants was determined via ELISA (R&D Systems, Minneapolis, MN, United States). The medium was renewed twice per week. Within 2 weeks of cell culture, CD11b+ cells differentiated from separate round shaped cells to a dense network of adherend cells (Supplementary Figure S1).

### Stimulation

After cultivation for 14 days, cells were stimulated for 24 h in medium with 5% FCS and 100 ng/ml LPS (*E. coli* O111:B4; Sigma Aldrich, Taufkirchen, Germany) or without LPS (control). Non-toxic concentration of LPS (100 ng/ml) has been titrated previously *in vitro* via MTT-assay. Similar LPS concentration has been used in other *in vitro* (Karlstetter et al., 2010, 2011) studies.

### Immunofluorescence

For immunofluorescent analysis the cells (6 × 10^4^/well) were cultured for 14 days on 8-well glass chamber slides (Merck Millipore). Then, cells were stimulated with/without LPS. After 24 h cells were fixed in PBS containing 4% paraformaldehyde (Carl Roth) for 30 min at room temperature (RT). Cells were air dried, unspecific binding sites were blocked by 5% goat serum (Biozol, Eching, Germany) in PBS for 1 h at RT. Cells were permeabilized using permeabilization buffer (Affymetrix, San Diego, CA, United States) in PBS/1% FCS for 30 min at RT, and stained using 0.5 μg/ml polyclonal goat anti-mouse/rat Iba1 (Abcam, Berlin, Germany) over night at 4°C. As secondary antibody 1 μg/ml polyclonal donkey anti-goat Alexa Fluor 488 (Abcam) were applied for 1 h at RT. Finally, nuclei were stained by 1 μg/ml Hoechst 33342 (Sigma Aldrich) and slides were mounted by using Mowiol (Sigma Aldrich). The cellular specimens were examined by immunofluorescence microscopy (ApoTome.2; Zeiss, Oberkochen, Germany) and displayed by ZEN 2.3 lite software (Zeiss).

### Cytokine and Chemokine Analysis

For cytokine analysis, cells were cultured in 6-well plates and were left unstimulated (control) or stimulated with LPS. The 24 h cell culture supernatants were harvested and analyzed for their cytokine content by ELISA: interleukin-6 (IL-6), tumor necrosis factor alpha (TNF-α), IL-10 – all purchased from Biolegend (Koblenz, Germany) and transforming growth factor beta 1 (TGF-β1; ELISA Ready-SET-Go Human/Maus TGF beta 1, eBioscience/Thermo Fisher, Dreieich, Germany). Reactions were performed in duplicates. Analysis was performed according to the manufacturer’s instructions.

For chemokine quantification including CC chemokine ligands (CCL) MCP-1 (CCL2), MIP-1α (CCL3), MIP-1β (CCL4), RANTES (CCL5), Eotaxin (CCL11), TARC (CCL17), MIP-3α (CCL20), and MDC (CCL22), C-X-C motif chemokine ligands (CXCL) KC (CXCL1), LIX (CXCL5), MIG (CXCL9), IP-10 (CXCL10), BLC (CXCL13), and bead-based multiplex LEGENDplex^TM^ analysis (LEGENDplex^TM^ Mouse Proinflammatory Chemokine Panel (13-plex; Biolegend) were used according to the manufacturer’s instructions. Reactions were performed in duplicates. Analysis was performed with the Cytoflex flow cytometer (Beckman Coulter, Krefeld, Germany). Data were analyzed via Legendplex V8.0 software (Biolegend) and specified as pg/ml.

### NO-Assay

Detection of NO was performed indirectly via nitrite in a 96-well flat bottom cell culture plate. 10 μM sodium nitrite standard (Sigma-Aldrich, Taufkirchen) was used 1:2 with PBS in a serial dilution series. Supernatants of cell culture were applied undiluted as a triple determination. Negative control (culture medium) was diluted 1:2 with PBS. To each well 50 μl of sulphanilic acid solution (1% sulphanilic acid (Sigma-Aldrich, Taufkirchen) in 4N HCL (Carl Roth, Karlsruhe) was added followed by 10 μl of concentrated HCL. The plate was incubated at RT for 10 min. 50 μl of N-(1-naphthyl) ethylenediamine solution (1% N-(1-naphthyl) ethylenediamine (Sigma-Aldrich) in methanol (Carl Roth) was added to each well. Intensity of discoloration was proportional to NO content in the sample. Measurement was performed with a wavelength of 550 nm in the microplate reader and NO content (in μM) was determined via linear regression.

### Flow Cytometry Analysis

For M1/2 phenotype classification via flow cytometry, cells were cultured in 6-well plates and were stimulated with LPS or left unstimulated (control). Following marker were used for M1/2 classification: M1 marker: CCR2; CD86; M2 marker: CX3CR1; CD206. For analysis of intracellular cytokines, brefeldin A (eBioscience) as a protein transport inhibitor was added for the last 6 h of 24 h stimulation, which promotes accumulation of intracellular cytokines within the cells. After 24 h, cells were harvested using Accutase (Biowest, Nuaillé, France), and prestained with CD16/CD32 Fc-block (93; TruStain fcX, Biolegend). The following antibodies were used for targeting: CD11b (BV510; M1/70), CX3CR1 (APC/Fire 750; SA011F11), CD206 (APC; C068C2), and CD86 (PE/Cy7; GL-1) – all purchased from Biolegend – and CCR2 (AF700; #475301) – purchased from R&D Systems (Wiesbaden, Germany). Cells were washed twice with PBS and were then analyzed.

For intracellular cytokine staining, cells were fixed with IC fixation buffer (eBioscience), permeabilized with permeabilization buffer (eBioscience), and incubated with one of the following anti-mouse antibodies targeting TNF-α (PE/Cy7; MP6-XT22), IL-6 (APC; MP5-20F3), IL-10 (PE/D 594; JES5-16E3), and TGF-β1 (PerCP/Cy5.5; TW7-16B4) – all purchased from Biolegend. Cells were washed twice with permeabilization buffer and analyzed. To exclude dead cells, cells were stained with the fixable viability dye (FVD) eFluor^®^ 450 (eBioscience) according to the manufacturer’s instructions.

Samples were measured using Gallios^TM^ 10/3 (Beckman Coulter) equipment. At least 300,000 viable FVD efluor^®^ 450-negative and CD11b-positive events per sample were counted using Kaluza 1.0 software. Data were analyzed using Kaluza Analysis 2.1 Software (Beckman Coulter) and the percentage of positive cells was documented.

### Statistics

For statistical analysis, GraphPad PRISM 7.04 software was used. The data obtained were analyzed for normal distribution (Shapiro-Wilk normally test) and for homogeneity of variance (Brown-Forsythe test). For multiple comparisons, a one-way ANOVA was performed. For a two-group comparison, an unpaired *t*-test was performed. Differences were considered as significant at ^∗^*p* < 0.05, ^∗∗^*p* < 0.01, ^∗∗∗^*p* < 0.001, and ^****^p < 0.0001.

## Results

### WT and IL-10 KO Iba1+ Cells *in vitro*

It is well known that microglia cells express CD11b which was used by Holt et al. for positive  MACS selection of brain derived microglia (Holt and Olsen, 2016). We first aimed to characterize the MACS-isolated CD11b-positive brain derived primary cells as microglia, and analyzed their morphological phenotype when left unstimulated (control) and upon pro-inflammatory conditions via LPS stimulation. As CD11b is not a microglia specific marker and other microglia specific marker as P2Y12 are down regulated upon LPS stimulation (Haynes et al., 2006) we used Iba1 as microglia related marker which has been widely used for detection of microglia both **in vitro** and **in situ** (Ito et al., 1998; Sukhorukova et al., 2012; Boche et al., 2013; Khozhai and Otellin, 2013; Sobin et al., 2013; Caldeira et al., 2014, 2017).

**FIGURE 1:**
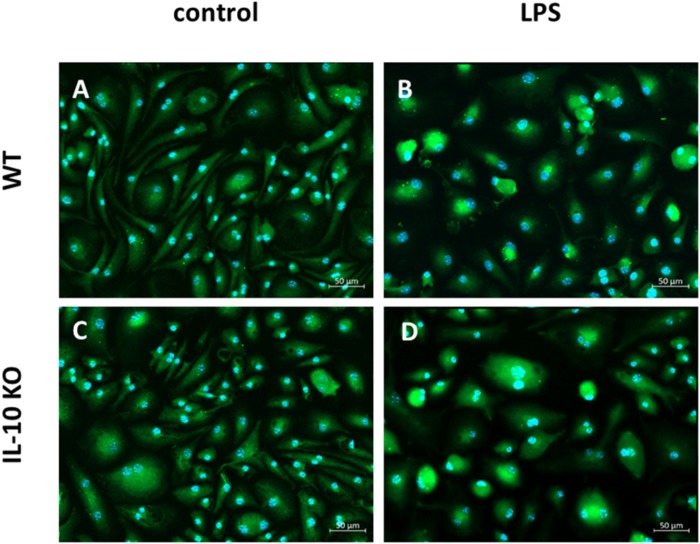
Fluorescence microscopy of Iba1+ WT and IL-10 KO microglia. **(A)** WT (control), **(B)** WT (LPS); slim cell bodies, **(C)** IL-10 KO (control), **(D)** IL-10 KO (LPS), round, amoeboid cell shape. Representative figures of four independent experiments (*n* = 4); overlay Iba1/green, nuclei/blue, 200-fold magnification.

Immunofluorescence staining of CD11b+ brain derived primary cells of WT and IL-10 KO mice against Iba1 showed that the majority of unstimulated and LPS stimulated cells from WT and IL-10 KO mice were positively stained for Iba-1. The mean fluorescence intensity of the Iba1+ staining did not differ between the culture conditions (data not shown). When left untreated Iba1+ cells showed slim cell bodies with elongated dendrites *in vitro* (Figures 1A,C). After 24 h stimulation with LPS, most of the Iba1+ cells showed a round cell shape with short cell extensions. This was the case for both WT and IL-10 KO Iba+ cells (Figures 1B,D). With regard to their origin, the isolation protocol, and the marker staining, the MACS sorted CD11b+ brain derived primary Iba1+ cells are termed as microglia cells.

### LPS Induced Cytokine and Chemokine Patterns of WT and IL-10 KO Microglia

**FIGURE 2:**
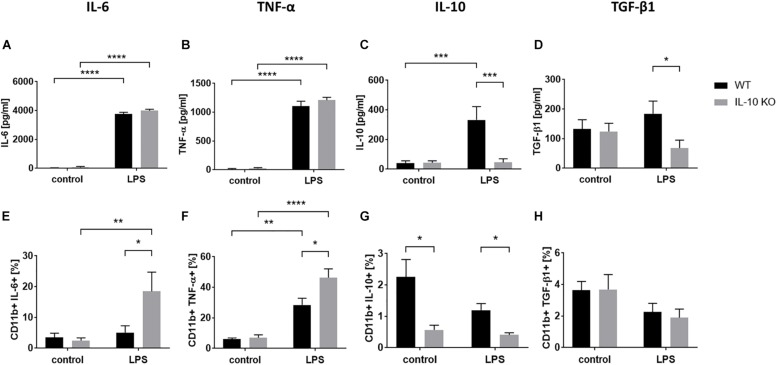
Cytokine pattern of WT (black bars) or IL-10 KO (gray bars) microglia of control group or after LPS treatment. ELISA data in pg/ml, **(A)** IL-6 (*N* = 11), **(B)** TNF-α (*N* = 11), **(C)** IL-10 (*N* = 10), and **(D)** TGF-β1 (*N* = 8), Mean ± SEM. Flow cytometric data in % CD11b+ cells, **(E)** IL-6, **(F)** TNF-α, **(G)** IL-10, **(H)** TGF-β1 (*N* = 5), mean ± SEM. One-way ANOVA, ^∗^*p* < 0.05; ^∗∗^*p* < 0.01; ^∗∗∗^*p* < 0.001; ^****^p < 0.0001.

Cytokine analysis via ELISA (Figures 2A–D and Supplementary Table S1) showed that LPS treatment led to significantly elevated secretion of IL-6 and TNF-α in the supernatants in both WT and IL-10 KO microglia (*p* < 0.0001). While IL-10 KO cells did not produce IL-10, WT cells secreted significantly more IL-10 upon LPS treatment than unstimulated cells (control: 40 ± 17 pg/ml, LPS: 332 ± 91 pg/ml; *p* < 0.001). Furthermore, IL-10 KO cells secreted a significantly lower amount of TGF-β1 upon LPS stimulation than WT cells (IL-10 KO: 68 ± 27 pg/ml; WT: 184 ± 43 pg/ml; *p* < 0.05).

Intracellular flow cytometry (Figures 2E–H and Supplementary Figure S2) showed that LPS stimulation significantly increased the number of TNF-α+ cells in both WT (control: 6 ± 0.8%, LPS: 28.5 ± 4.4%; *p* < 0.001) and IL-10 KO (control: 7 ± 1.9%, LPS: 46.4 ± 5.7%; *p* < 0.0001) microglia while the number of IL-6+ cells was only significantly increased in IL-10 KO (control: 2.5 ± 0.9%, LPS: 18.5 ± 6.2%; *p* < 0.001). Intracellular IL-10 staining was negative in IL-10 KO microglia and did not differ between the control and LPS group in WT microglia.

Overall, IL-10 KO microglia showed a significantly higher number of TNF-α- and IL-6-positive cells after LPS stimulation (*p* < 0.05) than WT microglia.

**FIGURE 3:**
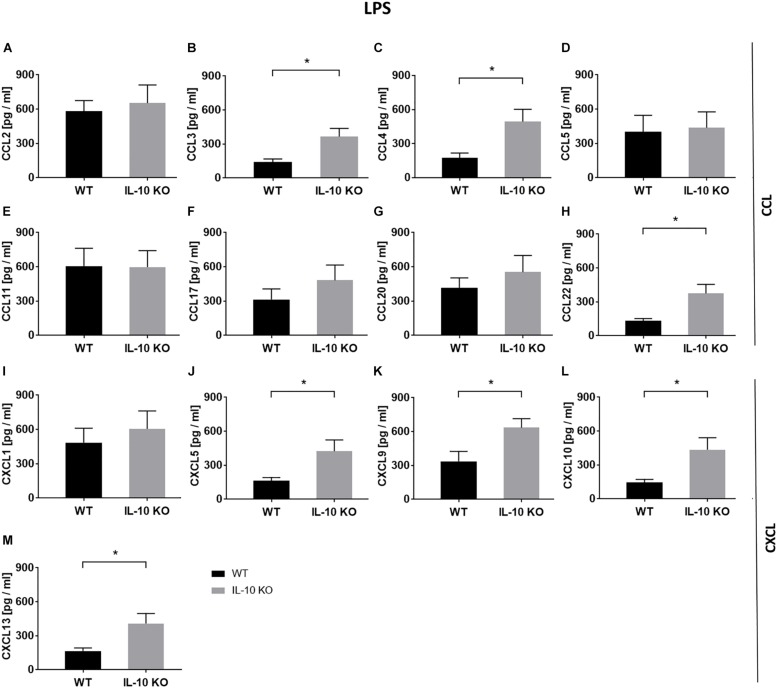
Chemokine release (Δ: LPS minus control) of WT (black bars) and IL-10 KO (gray bars) microglia under LPS treatment. LEGENDplex^TM^ data in pg/ml, **(A)** CCL2, **(B)** CCL3, **(C)** CCL4, **(D)** CCL5, **(E)** CCL11, **(F)** CCL17, **(G)** CCL20, **(H)** CCL22, **(I)** CXCL1, **(J)** CXCL5, **(K)** CXCL9, **(L)** CXCL10, and **(M)** CXCL13 (*N* = 8), mean ± SEM. Unpaired *t*-test, ^∗^*p* < 0.05.

In order to analyze chemokine patterns of WT and IL-10 KO microglia, supernatants of control and LPS-treated cells were analyzed according to their content of CCL and C-X-C motif chemokine ligands (CXCL) via LEGENDplex^TM^ analysis (Figure 3 and Supplementary Table S1). No differences were found between WT and IL-10 KO microglia cells in the control group (Supplementary Table S1). Compared to WT cells, IL-10 KO microglia showed a significantly enhanced net release (Δ = LPS minus control) of the CC family chemokines CCL3 (WT: 142 ± 26 pg/ml, IL-10 KO: 366 ± 71 pg/ml), CCL4 (WT: 175 ± 42 pg/ml, IL-10 KO: 494 ± 107 pg/ml), and CCL22 (WT: 133 ± 20 pg/ml, IL-10 KO: 375 ± 80 pg/ml) (Figures 3B,C,H; *p* < 0.05). This could also be observed for CXC family chemokines CXCL5 (WT: 163 ± 29 pg/ml, IL-10 KO: 425 ± 98 pg/ml), CXCL9 (WT: 337 ± 88 pg/ml, IL-10 KO: 635 ± 80 pg/ml), CXCL10 (WT: 145 ± 27 pg/ml, IL-10 KO: 436 ± 105 pg/ml), and CXCL13 (WT: 165 ± 28 pg/ml, IL-10 KO: 409 ± 87 pg/ml) (Figures 3J–M; *p* < 0.05).

Analysis of NO level in supernatants showed a significant increase upon LPS stimulation (*p* < 0.001) in supernatants of both, WT and IL-10 KO microglia without significant differences between both genotypes (Supplementary Figure S3).

### LPS Promotes M1 Phenotype in IL-10 KO Microglia

**FIGURE 4:**
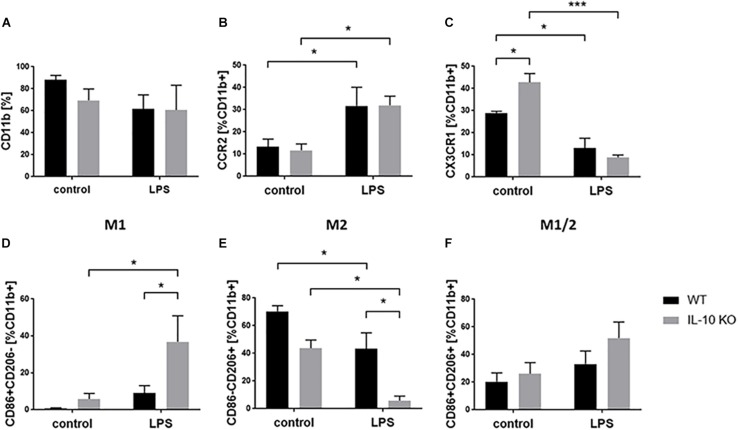
Flow cytometry of WT (black bars) and IL-10 KO (gray bars) microglia of control group or after LPS treatment. Data in% of CD11b+ cells, **(A)** CD11b, **(B)** CCR2, **(C)** CX3CR1, (D) M1 CD86+ CD206–, **(E)** M2 CD86-CD206+, **(F)** M1/2 CD86+ CD206+ (*N* = 3), mean ± SEM. One-way ANOVA, ^∗^*p* < 0.05; ^∗∗∗^*p* < 0.001.

Flow cytometry analyses of WT and IL-10 KO microglia showed that 88.01 ± 4.29% of WT and 69.34 ± 10.5% of IL-10 KO cells were CD11b+ in medium control. Expression of CD11b did not significantly changed upon LPS treatment to 61.54 ± 12.8% in WT and 60.54 ± 22.6% in IL-10 KO cells. Besides, no significant differences were detected between both genotypes (Figure 4A). Next, we checked the activation status of microglia by flow cytometry analysis, using CCR2 as a common M1 marker (Michelucci et al., 2009) and CX3CR1 as a marker for non-polarized microglia, respectively (Cunha et al., 2016). CCR2 was significantly upregulated after LPS stimulation in both WT (control: 13.4 ± 3.4%, LPS: 31.7 ± 8.4%; *p* < 0.05) and IL-10 KO (control: 11.8 ± 2.8%, LPS: 31.9 ± 4.1%; *p* < 0.05; Figure 4B) microglia without significant differences between the two genotypes. Baseline CX3CR1 expression was significantly higher in IL-10 KO microglia (42.8 ± 4%) than in WT microglia (29 ± 0.7%; *p* < 0.05; Figure 4C). Upon LPS stimulation CX3CR1 was significantly downregulated in both genotypes (WT: 13 ± 4.5%, *p* < 0.05; IL-10 KO: 8.8 ± 1.2%, *p* < 0.001) compared to the control groups.

M1/2 classification of microglia according to their expression of CD86 (M1) and CD206 (M2) (Zhou et al., 2017) showed no differences in the control group (Figures 4D–F). LPS treatment significantly elevated the number of CD86+ CD206− cells (M1 phenotype) in IL-10 KO microglia (control: 9 ± 4.1%, LPS: 36.9 ± 14%; *p* < 0.05; Figure 4D) while WT cells were unaltered. Both genotypes showed fewer CD86-CD206+ cells (M2 phenotype) after LPS treatment (WT: 43.8 ± 5.9%; IL-10 KO: LPS: 5.8 ± 3.4%; Figure 4E; *p* < 0.05), whereby these cells were significantly reduced in IL-10 KO cells compared to WT cells (Figure 4E; *p* < 0.05). Analysis of CD86+ CD206+ cells (intermediate M1/2 phenotype) did not show any differences upon LPS stimulation nor any differences related to the genotype, respectively (Figure 4F).

## Discussion

In this study we analyzed the effect of IL-10 deficiency on the M1/2 phenotype of microglia in an inflammatory environment *in vitro.* Herein, we isolated CD11b-positive cells from the brain of postnatal mice at the age of 2–14 days by using column-based magnetic separation with CD11b microbeads (Holt and Olsen, 2016).

As CD11b is not a microglia specific marker, cells were stained for microglia-related protein ionized calcium-binding adapter molecule 1 (Iba1) and subjected to immunofluorescence microscopy. Iba1 as microglia related marker which has been widely used for detection of microglia *in vitro* and *in situ* (Ito et al., 1998; Sukhorukova et al., 2012; Boche et al., 2013; Khozhai and Otellin, 2013; Sobin et al., 2013; Caldeira et al., 2014, 2017). Even though, both markers, CD11b and Iba1, are well established as common microglial markers (Robinson et al., 1986; Ito et al., 1998), they are not unique to microglia. However, other markers for microglia cells have been described e.g., TMEM119 (Bennett et al., 2016), Sall1 (Buttgereit et al., 2016), P2Y12 (Mildner et al., 2017), or Siglec-H (Konishi et al., 2017). But also these markers have drawbacks, as TMEM119 is absent in immature microglia (Satoh et al., 2016), Sall1 is not exclusively expressed by microglia during CNS development (Harrison et al., 2012; Buttgereit et al., 2016; Koso et al., 2018), and P2Y12 shows decreased expression in activated microglia (Haynes et al., 2006; Amadio et al., 2014; Mildner et al., 2017). Whereas the marker Siglec-H seem to be constitutively expressed in microglia indepenedently of their activation state (Konishi et al., 2017) suggesting this is a promising specific microglia marker. However, in the current study CD11b has been used for MACS based isolation of the primary brain derived microglia. As the CD11b expression was still high at time of sampling, we decided to use CD11b as a single pan marker for the gating of viable microglia in flow cytometry analysis. Other markers suitable for flow cytometric analysis of microglia are a combination of at least two markers e.g., CD11b^high^ CD45^med^ (Martin et al., 2017; Peng et al., 2017) and TEMEM119^+^ Sall1^+^ (Li et al., 2018).

In the current study we used the marker Iba-1 for characterization of microglia. Almost all cells of WT and IL-10 KO mice were positively stained for Iba1. Thus, with respect to the isolation method and origin of the cells we assume that these cells are brain derived microglia. Microglia were stimulated with LPS, which has been widely used to activate microglia both *in vitro* and *in vivo* (Becher and Antel, 1996; Nadeau and Rivest, 2000; Karlstetter et al., 2010, 2011). However, it has been previously shown that cultured microglia do not display the same dramatic change in morphology when activated (Kettenmann, 2006) as they do *in vivo*, where highly ramified microglia transform into amoeboid-phagocytic microglia (Hellwig et al., 2013; Torres-Platas et al., 2014). Thus, in the control group, the Iba1+ cells classified as microglia had long and slender cell bodies and showed a round-shaped amoeboid form upon LPS stimulation. These are the common morphological changes of microglia observed *in vitro* upon LPS stimulation (abd-el-Basset and Fedoroff, 1995). We did not perform further morphological analysis (e.g., ramification index, branches, spines soma diameter) (Jonas et al., 2012; Kozlowski and Weimer, 2012; Davis et al., 2017; Luckoff et al., 2017) in our cell culture model to evaluate morphological differences between WT and IL-10 KO microglia *in vitro*. However, it seems that lack of IL-10 did not influence the morphology of microglia dramatically when left untreated nor upon LPS treatment *in vitro*. Whereas, morphological analysis of spinal cord microglia *in situ* of LPS treated WT and IL-10 KO mice showed morphological differences in IL-10 KO mice (Anderson et al., 2017).

In order to examine the impact of IL-10 on the M1/2 phenotype of WT and IL-10 KO microglia, the cytokine and chemokine patterns of microglia upon LPS treatment were analyzed. According to their M1 or M2 phenotype activated microglia can exert different effector functions (Michelucci et al., 2009), and be either neurotoxic and generate a massive inflammatory response (M1 phenotype), releasing cytokines such as TNF-α and IL-6 (Magni et al., 2012), or dampen inflammation (M2 phenotype) by secreting anti-inflammatory mediators, including IL-10 and TGF-β (Suh et al., 2013; Tang and Le, 2016). At least it has to be assumed that the microglia phenotype is highly transitional and modulated in dependence on their environment (Goldmann and Prinz, 2013). Thus, we could show that both WT and IL-10 KO microglia express as well pro-inflammatory (IL-6, TNF-α, NO) and anti-inflammatory (WT: IL-10, TGF-β; IL-10 KO: TGF-β) cytokines pointing to a transitional phenotype including both M1 and M2 phenotype.

We could show that microglial activation by LPS enhanced secretion of IL-6 and TNF-α in WT and IL-10 KO cells. It is known that IL-10 downregulates the LPS-induced production of several proinflammatory cytokines (Frei et al., 1994; Lodge and Sriram, 1996). Elevated LPS induced IL-6 and TNF-α expression could be shown in brain tissue of IL-10 KO mice compared to WT mice via qPCR (Anderson et al., 2017). Therefore, we expected a higher amount of pro-inflammatory cytokine IL-6 and TNF-α in the supernatants of IL-10 KO microglia, but found no differences between WT and IL-10 KO cells. First, we assumed a saturation effect which make any differences undetectable via ELISA. Whereas, the flow-cytometric analysis showed that genetic depletion of IL-10 increased the frequency of IL-6+ and TNF-α+ cells after LPS stimulation. Which implies, that lack of IL-10 leads to an enhanced IL-6 and TNF-α expression upon LPS treatment.

When considering both, the similar cytokine content in both genotypes and the lower frequency of TNF-α+ and IL-6+ of WT cells, a higher cytokine release by WT cells upon LPS treatment could be also responsible for the similar cytokine content in the supernatant.

It has been previously shown that TGF-β1 plays a central role in microglial development and homeostasis both *in vitro* and *in vivo* (Butovsky et al., 2014). TGF-β1 is important in the microglial transition from a proinflammatory to a reparative M2 phenotype, by reducing microglial IL-6 secretion (Taylor et al., 2017). In an LPS-treated mixed culture of astrocytes and microglia, by microglia released IL-10 reduced the secretion of IL-1β, IL-6 and TNF-α (Ledeboer et al., 2000) and increased the release of TGF-β1 by astrocytes, which in turn attenuated the LPS-induced activation of microglia (Norden et al., 2014). In our study, IL-10 and TGF-β1 was upregulated in WT microglia after LPS stimulation, while IL-10 KO cells were negative for IL-10 and released significantly less TGF-β1. As TGF-b1 is important for the microglia homeostasis (Spittau et al., 2013), silencing of TGFb2R lead to an activated microglial phenotype and might prone the cells rather to a pro-inflammatory than an anti-inflammatory state (Zoller et al., 2018). Thus, IL-10 depletion in microglia cells might result in lower TGF-β1 release and thereby participate in regulating M2 cell frequency by influencing TGF-β1.

Microglia have been further shown to be potential sources of chemokines during CNS inflammation (Hesselgesser and Horuk, 1999) and that they upregulate chemokines at disease onset in EAE (Yamasaki et al., 2014). Chemokines represent a superfamily of small peptides, which act as chemoattractants to guide migration of other immune cells. Chemokines of the CXC (IP-10) and of the CC-family (MIP-1α, MIP-1β, MCP-1, RANTES) have been shown to be produced by microglia and may therefore contribute to the intracerebral recruitment of T cells, macrophages, and dendritic cells to the inflamed tissue (Aloisi, 2001). Previous studies showed an increased CCL2 secretion by primary microglia and astrocytes upon LPS stimulation *in vitro* (Hayashi et al., 1995), and *in vivo* thus mediating the migration of microglia, monocytes and lymphocytes to the sites of inflammation in the CNS (Gunn et al., 1997; Babcock et al., 2003). We confirmed the enhanced CCL2 release upon LPS stimulation in WT and IL-10 KO microglia, but could not show any impact of lacking IL-10 on the CCL2 release as shown for TGF-βR2 KO microglia *in vitro* where CCL2 was found to be enhanced secreted upon LPS treatment (Zoller et al., 2018).

In our study, LPS stimulation led to a significantly elevated release of chemokines of both the CC family CCL3 (MIP-1α), CCL4 (MIP-1β), and CCL22 (MDC) and the CXC family CXCL5 (LIX), CXCL9 (MIG), CXCL10 (IP-10), and CXCL13 (BLC) in IL-10 KO microglia compared to WT microglia. CCL3 and CCL4 are both members of the group of macrophage inflammatory proteins (MIP), which are important for inflammatory responses. It has been shown that they induce the synthesis and release of the proinflammatory cytokines IL-1, IL-6, and TNF-α from fibroblasts and macrophages (Irving et al., 1990; Ren et al., 2010). Moreover, microglia produce chemokines, for example, MDC, chemotactic for dendritic cells and differentiated T cells during EAE or after *in vitro* exposure to IFN-γ (Aloisi, 2001). Thus, an enhanced release of chemokines by microglia in pro-inflammatory environment might support the development of a more severe EAE and an accelerated disease progression in IL-10 KO mice (Bettelli et al., 1998; Samoilova et al., 1998; Anderson et al., 2004; Zhang et al., 2004). With regard to the improvement of therapeutic strategies it might be more effective to change the M1 or M2 phenotype of resident microglia, than to influence the peripheral immune response via systemic drugs. It has been already shown that M2 polarization of macrophages or microglia is a promising treatment option in EAE (Butovsky et al., 2006; Mikita et al., 2011; Cao and He, 2013; McMurran et al., 2016). Favoring M2 generation or respective modulation of microglia might have beneficial effects in chronic diseases as shown for IL-10 in the EAE model (Yang et al., 2009). Furthermore, modulation of retinal microglia via specific ligands (e.g., TSPO, minocycline) has been shown to be beneficial for the outcome of retinal degenerative diseases (Akhtar-Schafer et al., 2018).

The differential M1 or M2 activation states of microglia coexist in inflamed tissues during the development of neurodegenerative diseases and can mediate cell damage or neuroprotective effects. M1 cells can induce tissue damage, demyelination, and neuronal death in the CNS in the EAE model (Almolda et al., 2011; Liu et al., 2013; Jiang et al., 2014), whereas M2 cells are known to be involved in suppressing EAE (Jiang et al., 2014) and in resolving inflammation and repairing tissue (Laria et al., 2016).

IL-10 may counteract an immune response toward an M1 phenotype and modulate the polarization of microglial cells to a more anti-inflammatory M2 phenotype, likewise, to control neuroinflammation.

To further analyze the microglia phenotype we used the surface marker CCR2 as M1 marker (Michelucci et al., 2009) and CX3CR1 as a marker for non-polarized microglia, respectively (Cunha et al., 2016). Previous studies showed an increased CCL2 secretion and/or CCR2 expression *in vitro* and *in vivo* during pro-inflammatory conditions (Hayashi et al., 1995; Berman et al., 1996; Gunn et al., 1997; Babcock et al., 2003; Glabinski et al., 2003). Our results confirmed the increased CCL2 and CCR2 expression upon LPS stimulation in WT and IL-10 KO cells without significant difference between both genotypes.

We further studied the fractalkine receptor CX3CR1, which is highly expressed in the non-polarized M0 microglial phenotype (Sheridan and Murphy, 2013). As previously shown for N9 microglial cells (Cunha et al., 2016), we showed that CX3CR1 was significantly downregulated after LPS stimulation in both WT and IL-10 KO microglia.

Lively et al. describe a IL-10 mediated down regulation of CXCR1 expression in murine primary microglia (Lively et al., 2018). We can confirm this finding as the frequency of CX3CR1+ microglia was higher in unstimulated IL-10 KO microglia than in WT microglia, indicating a suppressive effect of IL-10 on CX3CR1 expression.

Classification via the marker CCR2 and CX3CR1 used for macrophages did not show differences in the two genotypes uponLPS treatment. We therefore include further M1 (CD86) and M2 (CD206) marker which were used for M1/2 phenotyping of microglia cells (Peng et al., 2017; Zhou et al., 2017). Here, our analysis showed a reduced M2 CD86-CD206+ phenotype and an unaltered M1/2 CD86+ CD206+ phenotype in both genotypes upon LPS treatment. However, the M2 phenotype was significantly reduced in IL-10 KO compared to WT microglia after LPS stimulation. Furthermore, an LPS-induced M1 CD86+ CD206− phenotype could be shown in IL-10 KO cells, but not in WT cells.

Taken together, our results suggest that IL-10 deficiency did not have a significant effect on microglia M1/2 phenotype when left untreated. Whereas lack of IL-10 promotes microglia to an increased expression of pro-inflammatory cytokines/chemokines and the lower release of TGF-β1 as anti-inflammatory cytokine. Thus, these results indicate that absence of IL-10 led to a M1-prone microglial phenotype during LPS treatment.

## Data Availability Statenment

The datasets generated for this study are available on request to the corresponding author.

## Ethics Statement

The animal experiments were performed in accordance with the European Health Law of the Federation of Laboratory Animal Science Associations (FELASA) and regulations of the Society for Laboratory Animal Science (GV-SOLAS) in Germany. The protocol was approved by the North Rhine-Westphalia State Agency for Nature, Environment, and Consumer Protection (LANUV) (authorization number AZ 2015.A011/§4.16.006). All experimental procedures conformed to the guidelines of the National Institutes of Health and to the Association for Research in Vision and Ophthalmology resolution on the use of animals in research.

## Author Contributions

BL, MK, DB, ST, GM, TL, and AH designed the study and wrote the manuscript. BL, DB, SW, MB, TJ, and MK performed the experiments and analyzed the data. All authors read and approved the final version of the manuscript.

## Conflict of Interest

The authors declare that the research was conducted in the absence of any commercial or financial relationships that could be construed as a potential conflict of interest.

## Supplementary Material

FIGURE S1 Isolated CD11b+ cells cultured in 8-well chamber slides develop from **(A)** round shaped cells at day 1 to cells with growing cell extension at **(B)** day 4, **(C)** day 7, and **(D)** after 12 days in cell culture to a dense network of cells (Magnification ×200).

FIGURE S2 Gating Strategy for primary microglia in flow cytometry. **(A)** Microglia in SSC/FSC profile, **(B)** microglia stained with the live-dead dye eFluor450. Living cells (FVD eFlour450 negative) are shown in region 1 (R1), **(C)** isotpye control IgG2b κ BV510 and IgG1 κ APC, **(D)** CD11b+ BV510 cells with IgG1 κ APC Isotype control, and **(E)** intracellular cytokine staining of CD11b+ cells with anti-IL-6 APC.

FIGURE S3 NO release of WT (black bars) or IL-10 KO (gray bars) microglia of control group or after LPS treatment. NO content in μM (*N* = 12) in the supernatant of microglia. Mean ± SEM. One-way ANOVA. ^****^*p* < 0.0001.

TABLE S1 Cytokine/Chemokine level.

## Abbreviations

CCL: chemokine (C-C motif) ligand
CD: cluster of differentiation
CNS: central nervous system
CX3CL: chemokine (C-X3-C motif) ligand
EAE: experimental autoimmune encephalomyelitis
ELISA: enzyme-linked immunosorbent assay
IFN- γ: interferon gamma
IL: interleukin
KO: knock out
LPS: lipopolysaccharide
M-CSF: macrophage colony-stimulating factor
MS: multiple sclerosis
TGF- β: transforming growth factor beta
TNF- α: tumor necrosis factor-alpha
WT: wild type.
