# Supplementary figures and images for: Loss of IL-10 Promotes Differentiation of Microglia to a M1 Phenotype
Source: Front Cell Neurosci. 2019 Oct 9;13:430. doi: 10.3389/fncel.2019.00430 (PMC6794388; doi:10.3389/fncel.2019.00430)

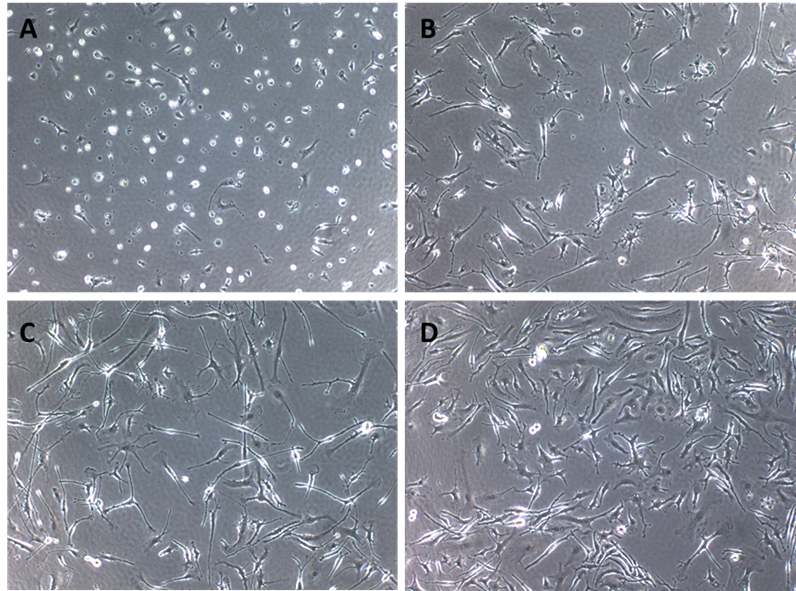

Supplement: FIGURE S1 — Isolated CD11b+ cells cultured in 8-well chamber slides develop from (A) round shaped cells at day 1 to cells with growing cell extension at (B) day 4, (C) day 7, and (D) after 12 days in cell culture to a dense network of cells (Magnification ×200). [file Image_1.TIF]

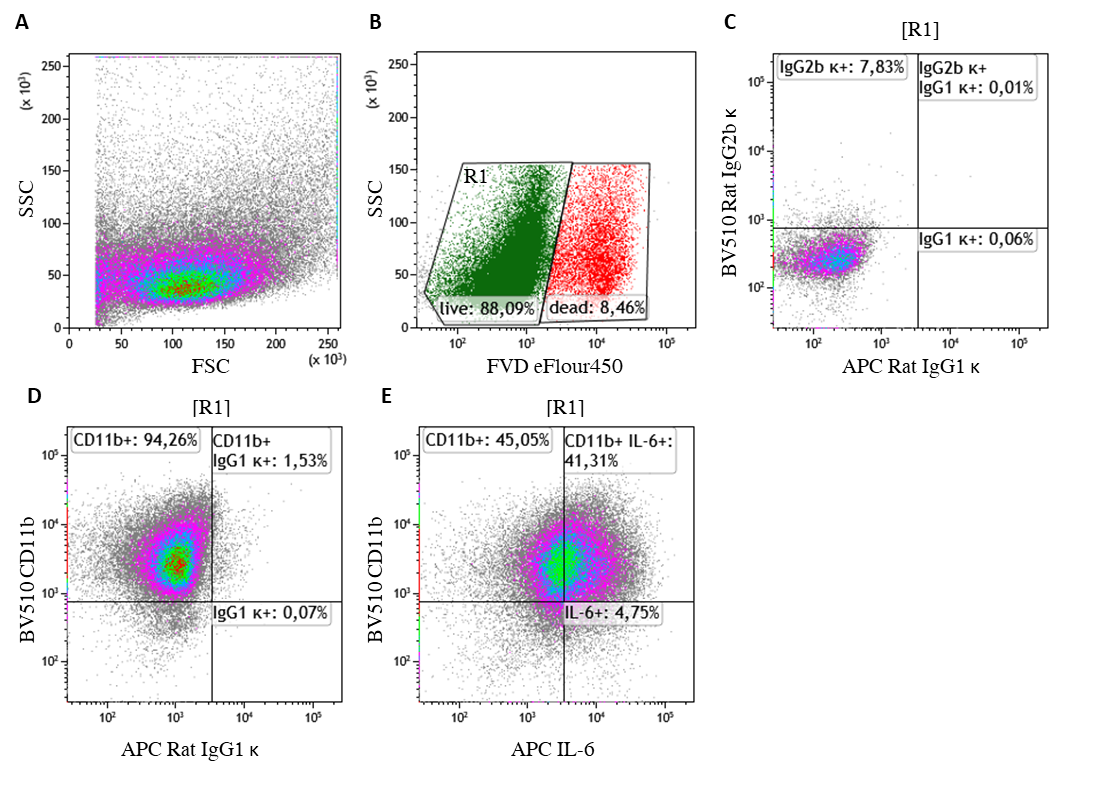

Supplement: FIGURE S2 — Gating Strategy for primary microglia in flow cytometry. (A) Microglia in SSC/FSC profile, (B) microglia stained with the live-dead dye eFluor450. Living cells (FVD eFlour450 negative) are shown in region 1 (R1), (C) isotpye control IgG2b κ BV510 and IgG1 κ APC, (D) CD11b+ BV510 cells with IgG1 κ APC Isotype control, and (E) intracellular cytokine staining of CD11b+ cells with anti-IL-6 APC. [file Image_2.TIF]

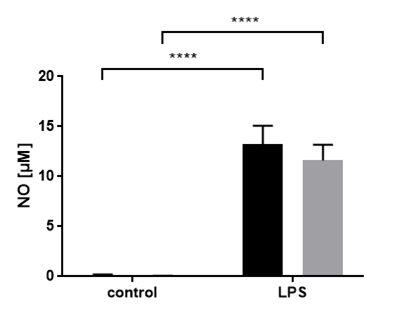

Supplement: FIGURE S3 — NO release of WT (black bars) or IL-10 KO (gray bars) microglia of control group or after LPS treatment. NO content in μM (N = 12) in the supernatant of microglia. Mean ± SEM. One-way ANOVA. ****p < 0.0001. [file Image_3.TIF]
