# Supplementary material for: Loss of IL-10 Promotes Differentiation of Microglia to a M1 Phenotype
Source: Front Cell Neurosci. 2019 Oct 9;13:430. doi: 10.3389/fncel.2019.00430 (PMC6794388; doi:10.3389/fncel.2019.00430)
Supplement: TABLE S1 — Cytokine/Chemokine level. [file Table_1.docx]

**Supplemental Table 1.** Cytokine / Chemokine level

|  | **WT** | | | **IL-10 KO** | | |
| --- | --- | --- | --- | --- | --- | --- |
| **Protein** | **Control** | **LPS** | **p<** | **Control** | **LPS** | **p<** |
| IL-6 | 23.3 ± 13.7 | 3767.0 ± 117.6 | 0.0001 | 72.5 ± 60.7 | 3992.0 ± 103.4 | 0.0001 |
| TNF-α | 11.8 ± 11.1 | 1103.0 ± 87.6 | 0.0001 | 23.5 ±14.2 | 1208.0 ± 49.3 | 0.0001 |
| IL-10 | 40.1 ± 16.9 | 332.0 ± 90.9 | 0.001 | 42.9 ± 13.6 | 45.3 ± 25.1 | >0.9 |
| TGF-β1 | 132.5 ± 31.4 | 184.3 ± 42.7 | 0.47 | 124.2 ± 27.9 | 67.9 ± 27.3 | 0.42 |
| CCL2 | 43.4 ± 19.0 | 626.9 ± 86.3 | 0.001 | 54.4 ± 19.1 | 708.3 ± 154.2 | 0.0001 |
| CCL3 | 12.2 ± 2.4 | 154.3 ± 25.1 | 0.05 | 15.4± 6.9 | 381.7 ± 74.6 | 0.0001 |
| CCL4 | 12.1 ± 2.3 | 187.0 ± 40.9 | 0,08 | 9.3 ± 1.6 | 503.4 ± 108.7 | 0.001 |
| CCL5 | 38.6 ± 16.9 | 442.0 ± 153.0 | 0.05 | 40.3 ± 16.0 | 478.2 ± 137.8 | 0.05 |
| CCL11 | 43.6 ± 19.6 | 648.6 ± 164.1 | 0.01 | 36.0 ± 17.7 | 633.7 ± 146.3 | 0.01 |
| CCL17 | 33.5 ± 15.2 | 348.3 ± 101.9 | 0.05 | 39.8 ± 19.2 | 522.0 ± 139.7 | 0.01 |
| CCL20 | 42.4 ± 20.9 | 459.3 ± 97.3 | 0.05 | 35.1 ± 16.5 | 588.9 ± 154.1 | 0.001 |
| CCL22 | 12.0 ± 1.9 | 144.6 ± 18.9 | 0,07 | 11.4 ± 3.2 | 386.6 ± 82.0 | 0.0001 |
| CXCL1 | 46.5 ± 24.0 | 532.2 ± 137.9 | 0.05 | 42.8 ± 18.6 | 648.5 ± 163.2 | 0.01 |
| CXCL5 | 12.9 ± 2.3 | 175.4 ± 28.2 | 0,07 | 10.2 ± 1.9 | 435.3 ± 100.1 | 0.0001 |
| CXCL9 | 23.4 ± 7.8 | 360.6 ± 85.4 | 0.01 | 19.2 ± 10.7 | 653.9 ± 84.5 | 0.0001 |
| CXCL10 | 14.3 ± 2.6 | 159.7 ± 25.6 | 0.14 | 12.9 ± 3.2 | 449.3 ± 106.9 | 0.01 |
| CXCL13 | 13.1 ± 2.5 | 178.2 ± 27.0 | 0.05 | 10.8 ± 2.0 | 420.0 ± 88.5 | 0.0001 |
| Cytokine / Chemokine release of WT and IL-10 KO microglia in the supernatant of medium control or after 24h Medium (control) and LPS treatment. Data in mean ± SEM [pg/ml] (Cytokine N=11 / Chemokine N=8). P values shown for control vs. LPS (one-way ANOVA). | | | | | | |
